# Gene variants associated with skin barrier dysfunction in atopic dermatitis: a systematic review and meta-analysis

**DOI:** 10.1590/1984-0462/2025/43/2025111

**Published:** 2025-12-12

**Authors:** Priscila de Lima Cordeiro, Caroline Guth de Freitas de Moraes, Lilian Pereira Ferrari, Nelson Augusto Rosário

**Affiliations:** a Universidade Federal do Paraná, Curitiba, PR, Brazil.

**Keywords:** Atopic dermatitis, FLG; SPINK5, Genetic variants, Skin barrier, Barreira cutânea, Dermatite atópica, FLG, SPINK5, Variantes gênicas

## Abstract

**Objective:**

The aim of this systematic review and meta-analysis was to identify genetic variants associated with skin barrier dysfunction and analyze their contribution to the development of atopic dermatitis (AD).

**Data source:**

A comprehensive search of six databases (2002–2022) yielded 20 eligible casecontrol studies involving European and Asian populations.

**Data synthesis:**

Meta-analyses revealed significant associations between AD and specific variants in the *FLG, SPINK5, LAMA3, HRNR*, and *COL8A1* genes. Notably, *FLG* variants such as R501X, 3321delA, and rs61816761 showed high odds ratios (up to OR=11.22), particularly in Spanish and Korean populations. *SPINK5* variants including A1103G and G1258A were also significantly associated, especially in Asian cohorts.

**Conclusions:**

Variants affecting skin barrier integrity are strongly linked to AD susceptibility. These findings confirm the role of genetic factors across diverse populations and support translational strategies such as genetic screening, early diagnosis, and personalized treatment in pediatric dermatology.

## RESUMO

**Objetivo:**

Esta revisão sistemática com metanálise teve como objetivo identificar variantes genéticas associadas à disfunção da barreira cutânea e analisar sua contribuição para o desenvolvimento da dermatite atópica (DA).

**Fontes de dados:**

Uma busca abrangente em seis bases de dados (2002–2022) resultou na inclusão de 20 estudos caso-controle envolvendo populações da Europa e da Ásia.

**Síntese dos dados:**

As metanálises revelaram associações significativas entre a DA e variantes específicas dos genes *FLG, SPINK5, LAMA3, HRNR* e *COL8A1*. Destacam-se as variantes do *FLG,* como R501X, 3321delA e rs61816761, com razões de chances (OR) elevadas (até OR=11,22), especialmente em populações da Espanha e Coreia do Sul. As variantes do *SPINK5*, como A1103G e G1258A, também apresentaram associação significativa, principalmente em coortes asiáticas.

**Conclusões:**

Variantes genéticas que afetam a integridade da barreira cutânea estão fortemente associadas à suscetibilidade à DA. Os achados confirmam o papel dos fatores genéticos em diferentes populações e reforçam estratégias translacionais, como o rastreamento genético, o diagnóstico precoce e o tratamento personalizado na dermatologia pediátrica.

## INTRODUCTION

Atopic dermatitis (AD), also known as atopic eczema, is a chronic and recurrent inflammatory skin disease that affects both adults and children, with a prevalence rate of 1–3% in adults and over 20% in children.^1,2^ AD is characterized by recurrent eczema, xerosis, intense pruritus, and lichenification. It is a heterogeneous disease that varies with age, geographic location, and phenotype. A clinical AD diagnosis is based on evaluations of skin lesions, personal and family atopic history, and other clinical symptoms; Hanifin and Rajka criteria are considered as the gold standard for diagnosis.^3,4^

The pathogenesis of AD is multifactorial, involving complex interactions between genetic predisposition, immune dysregulation, environmental exposures, and dysfunction in both the skin barrier and microbiota.^5,6^ A compromised skin barrier facilitates transepidermal water loss and allows the penetration of allergens and microorganisms, perpetuating inflammation.^6,7^ Recent evidence highlights the contribution of epigenetic mechanisms, including DNA methylation, histone modifications, and microRNAs, in regulating gene expression in AD, particularly in response to environmental factors such as air pollution and smoking.^5,8^

In parallel, studies have emphasized the involvement of the skin and gut microbiota in AD pathogenesis, reinforcing the relevance of the gut-skin axis and its role in immune modulation. Metabolites produced by microbiota, such as short-chain and conjugated fatty acids, help to regulate inflammatory processes and enhance skin barrier integrity, pointing to potential new therapeutic strategies.^9^

Beyond its biological underpinnings, AD is also a relevant public health concern due to its negative impact on quality of life, frequent comorbidities, including sleep disturbances and psychiatric conditions, and substantial social and economic burden. These aspects highlight the importance of advancing knowledge and improving the integrated management of the disease.^10^

Children with AD are at a higher risk of developing asthma and allergic rhinitis, a phenomenon known as the atopic march, which suggests that children with AD may present with other atopic diseases during childhood.^1,11^

At least 46 genes are associated with AD risk, including loss-of-function variants in the filaggrin gene (*FLG*) on chromosome 1q21.3, which has been implicated in epidermal differentiation and skin barrier function. Other genes that affect epidermal differentiation and cutaneous and systemic immunity influence AD pathogenesis. Genome-wide association studies (GWAS) have identified more than 30 loci related to skin barrier function, immunological dysfunction, and innate immune signaling.^1,12^ More recent GWAS and transcriptome-wide association study (TWAS) have identified over 100 risk loci for AD, many of which are related to skin barrier integrity and immune response pathways, with variation observed among different ancestral populations.^13,14^

These advances in genetic and molecular understanding support the implementation of precision medicine strategies focused on early risk stratification, personalized treatment, and disease prevention in AD.^13,15^ Nevertheless, despite these discoveries, no prior meta-analysis has integrated global data with an emphasis on genetic variants specifically associated with skin barrier dysfunction. This systematic review and meta-analysis aimed to identify and assess the impact of such variants on AD pathophysiology. By compiling data from diverse populations, this study offers a robust synthesis that informs future diagnostic and therapeutic approaches.

## METHOD

This systematic literature review followed the 2020 Preferred Reporting Items for Systematic Reviews and Meta-Analyses (PRISMA) guidelines checklist^16^ and was registered in the International Prospective Register of Systematic Reviews (ID CRD42022322213).

The search covered articles published between 2002 and 2022 on genes associated with skin barrier dysfunction in AD; six databases with specific search strategies were utilized. The following search strategies were utilized to identify relevant studies for our review. Our primary keywords were “atopic dermatitis,” “genetics,” and “skin barrier,” and their associated terms, according to Table 1.

**Table 1.** Search strategies.

Database	Search strategies
BVS	((Dermatite Atópica) OR (Dermatitis, Atopic) OR (Dermatitis Atópica)) AND ((Barreira Cutânea) OR (Barreira epidérmica) OR (Barreira epitelial) OR (Skin barrier) OR (Epithelial barrier) OR (Epidermal barrier) OR (barrera de la piel) OR (barrera epitelial) OR (barrera epidérmica)) AND ((genética) OR (genetics) OR (genes) OR (técnicas genéticas) OR (genetic techniques) OR (fen“menos genéticos) OR (genetic phenomena) OR (fenómenos genéticos))
PubMed	(((“Dermatitis, Atopic”[Mesh]) OR (atopic dermatitis[Title/Abstract])) AND (((skin barrier[Title/Abstract]) OR (epithelial barrier[Title/Abstract])) OR (epidermal barrier[Title/Abstract]))) AND (((((“Genetics”[Mesh]) OR “Genes”[Mesh]) OR “Genetic Techniques”[Mesh]) OR “Genetic Phenomena”[Mesh]) OR ((((genetics[Title/Abstract]) OR (genes[Title/Abstract])) OR (genetic techniques[Title/Abstract])) OR (genetic phenomena[Title/Abstract])))
Embase	‘atopic dermatitis’:ti,ab,kw AND (‘skin barrier’:ti,ab,kw OR ‘epithelial barrier’:ti,ab,kw OR ‘epidermal barrier’:ti,ab,kw) AND (genetics:ti,ab,kw OR gene:ti,ab,kw OR ‘genetic procedures’:ti,ab,kw OR heredity:ti,ab,kw)
CINAHL	TX atopic dermatitis AND TX (skin barrier OR epithelial barrier OR epidermal barrier) AND TX (genetics OR genes OR genetic procedures OR heredity)
Scopus	(TITLE-ABS-KEY (atopic,dermatitis)) AND ((TITLE-ABS-KEY (skin,barrier) OR TITLE-ABS-KEY (epithelial,barrier) OR TITLE-ABS-KEY (epidermal,barrier))) AND ((TITLE-ABS-KEY (genetics) OR TITLE-ABS-KEY (gene) OR TITLE-ABS-KEY (genetic,techniques) OR TITLE-ABS-KEY (genetic,phenomena) OR TITLE-ABS-KEY (genetic,procedures) OR TITLE-ABS-KEY (heredity)))
Web of Science	(atopic dermatitis) (Tópico) and (((skin barrier)) OR (epithelial barrier)) OR (epidermal barrier) (Tópico) and ((((((genetics)) OR (genes)) OR (genetic techniques)) OR (genetic phenomena)) OR (genetic procedures)) OR (heredity) (Tópico)

BVS: Biblioteca Virtual em Saúde; CINAHL: Cumulative Index to Nursing and Allied Health Literature.

The review comprised case-control studies that included patients diagnosed with AD and healthy controls without an allergic history, addressing genetic variants associated with skin barrier dysfunction, and were published in English or Portuguese. Excluded studies were those for animals, in vitro experiments, or other skin diseases; those that did not report the allergy history for the controls; and those that reported genes addressing different aspects, such as the immune response.

Controls in the included studies were selected based on the absence of any personal history of allergic diseases, including AD, asthma, allergic rhinitis, and food allergy, to minimize overlap or delayed-onset AD cases.

Two reviewers (PLC and CGM), independently and together, performed all procedures: screening, full-text reading, selection, and data collection; disagreements were resolved by a third reviewer (LPF). The included articles were selected based on the acceptance of at least two of the three reviewers. The Rayyan platform (https://www.rayyan.ai/) was used to exclude duplicates and screening the studies. Microsoft Office Excel (Microsoft Corporation, Redmond, WA, USA) was used for data collection. The tabulated information included location, number of participants, sex, age, severity classification, comorbidities associated with AD, and genes and their respective variants. The Newcastle-Ottawa Scale (NOS) was selected because it is a validated and widely used tool for assessing the risk of bias in observational studies, evaluated independently and in pairs.

This study used quantitative and qualitative variables to analyze data, such as age, symptom severity, and associated phenotypes, using mean (± standard deviation) for quantitative variables and absolute number (percentage of the total) for qualitative variables.

For the meta-analysis, the number of events in the disease and control groups and the total number of patients in the disease and control groups were extracted from the studies. Using the “meta” package in R, effect size information was extracted through the odds ratios (ORs), confidence intervals (CIs), and heterogeneity between studies.^17^

As a significant variation between studies was expected, a random-effects model was used to pool the effect sizes. The heterogeneity variance (tau^2^) was calculated using the Sidik– Jonkman estimator. Studies without controls or disease cases were excluded because no continuity correction method for zero values was applied. An influence analysis and outlier detection were performed, and the outliers were removed.

The Baujat diagnostic plot was used to validate the relationship between heterogeneity and its influence on the results. Forest plots were used to visualize effect sizes for the meta-analysis results.

A subgroup analysis was conducted to better understand the variation in the results. Specifically, the studies were grouped by continent, separating them into European and Asian subgroups. This approach allowed the assessment of whether geographical differences influenced the observed effect sizes, providing a more detailed and contextualized view of the data.

We considered stratification by other variables (age, sex, severity, and comorbidities), but such subgroup analyses were not feasible due to a lack of raw data; studies provided only summarized demographic data. All analyses and graphs were created using R statistical software.^18^

## RESULTS

**Figure 1.**
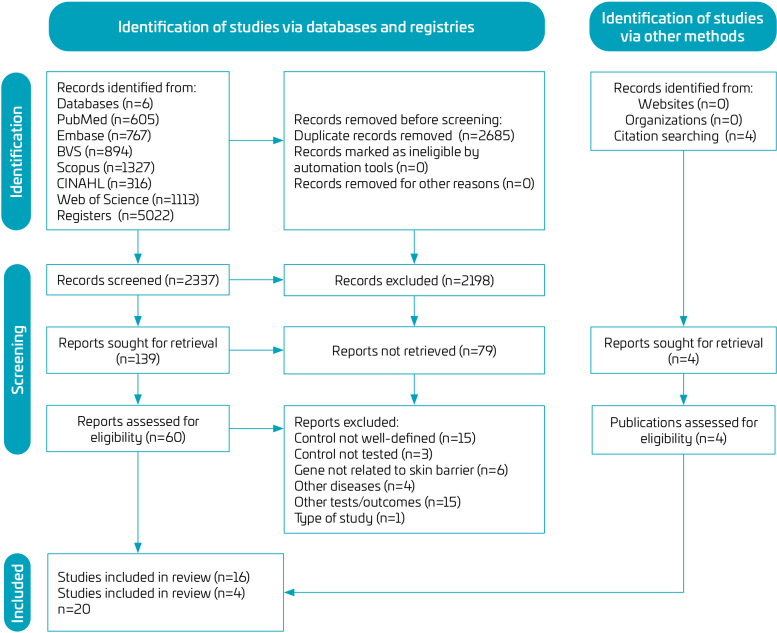
Preferred Reporting Items for Systematic Reviews and Meta-Analyses flow diagram.

BVS: Biblioteca Virtual em Saúde; CINAHL: Cumulative Index to Nursing and Allied Health Literature.

Per PRISMA guidelines, a flow diagram was generated (Figure 1). A search in the six databases resulted in 5022 articles. After removing duplicates, 2337 articles were screened by title and abstract, and 139 articles were selected for full-text reading. Of these, eligibility criteria were applied to 60 articles, resulting in 16 acceptable articles. After an additional search of their bibliographies, four articles were obtained; thus, 20 articles were included in the subsequent stages.

The studies included in this meta-analysis are described in references from 19 to 38, and their characteristics are presented in Table 2.^19,20,21,22,23,24,25,26,27,28,29,30,31,32,33,34,35,36,37,38^

**Table 2.** Characteristics of the studies.

Reference	Local	Sample size (patients/controls)	Mean age (patients/controls), years	Gene (Variant)
Trzeciak et al.^19^	Poland	159/108	24.8/27.1	CRNN (rs941934); RPTN (rs28441202, rs3001978, rs12117644)
Rasool et al.^20^	Kashmir	100/106	33.3/35.3	FLG (R501X, 2282del4); LELP-1 (rs7534334)
Zhao et al.^21^	China	91/250	ND/27	SPINK5 (A1103G, G1156A, G1258A, G2475T)
Kato et al.^22^	Japan	124/110	ND	SPINK5 (IVS12–26C>T, 1188T>C, 1103A>G, 1156G>A, 1258G>A)
Stemmler et al.^23^	Germany	470/320	19/62	LAMA3 5’UTR ANKRD29 (rs7238623, rs8096061, rs1613739); LAMA3 (rs12960692, rs8083184, rs1711450, etc.); LAMB3 (rs2566, rs2009292, rs3179860, etc); LAMC2 (rs483783, rs601508, rs2274980, rs11586699)
Strafella et al.^24^	Mediterranean	428/1042	ND	COL6A5 (rs12488457); COL8A1 (rs13081855); COL10A1 (rs3812111)
Klonowska et al.^25^	Poland	239/170	ND	TSLP (rs1898671); FLG (2282del4, R501X)
Lesiak et al.^26^	Poland	163/204	ND	FLG (R501X, 2282del4)
Jurakic Toncic et al.^27^	Croatia	100/50	ND	FLG (R501X, 2282del4, R2447X)
Dębińska et al.^28^	Poland	87/71	1.10/1.27	FLG (R501X, 2282del4, R2447X, S3247X)
Enomoto et al.^29^	Japan	376/923	ND	FLG (3321delA, S2554X, rs2065958, rs3814299, rs12730241)
Dębińska et al.^30^	Poland	103/85	1.10/1.27	HRNR (rs877776); FLG2 (rs12568784); FLG (R501X, 2282del4, R2447X, S3247X)
Yu et al.^31^	South Korea	1430/862	5.17/9.47	FLG (3321delA, E2422X, R501X)
Zhang et al.^32^	China	261/92	7.11/16.49	FLG (K4671X, 3321delA)
Churnosov et al.^33^	Russia	700/612	42.73/42.56	FLG (rs12130219, rs558269137, rs61816761, rs77199844, etc.)
Ercan et al.^34^	Turkey	49/50	4.85/3.77	FLG (R501X)
González-Tarancón et al.^35^	Spain	111/103	5.51/9.72	FLG (R501X, 2282del4, R2447X)
Yoon et al.^36^	South Korea	279/224	13.7/24.6	KLK7 (3’UTR AACC ins5874); FLG (3321delA, pK4022X); SPINK5 (1156G>A, 2475G>T, 1188T>C)
Dežman et al.^37^	Slovenia	241/164	23.5/41.7	SPINK5 (rs2303067)
Woźniak et al.^38^	Poland	60/61	ND	FLG (R501X, 2282del4)

Source: The author, 2023.

Notes: Sample size and mean age are presented as patients/controls.

ND: no data.

For studies reporting multiple SNPs, only some variants are listed in the table to optimize space; full details are available in the original publications.

All the included articles involved European and Asian populations. Of these, 60% were from Europe (Poland, Germany, the Mediterranean region, Croatia, Russia, Spain, and Slovenia) and 40% from Asia (Kashmir, China, Japan, South Korea, and Turkey). Thirteen articles reported symptom severity, with a prevalence of the mild form of the disease; 12 mentioned allergic comorbidities, most frequently asthma.

Table 3 shows that the methodological quality of the case-control studies included in this review was carefully assessed using the NOS.

**Table 3.** Risk of bias and methodological quality assessment using the NOS Scale.

Reference	Selection				Comparability	Exposure			Score
	1	2	3	4	1	1	2	3	
Trzeciak et al.^19^	*	*	*	*	**	*	*		8/9
Rasool et al.^20^	*	*	*	*	**	*	*		8/9
Zhao et al.^21^	*	*	*	*	**	*	*		8/9
Dębińska et al.^30^	*	*	*	*	**	*	*		8/9
Strafella et al.^24^	*	*	*	*		*	*		6/9
Klonowska et al.^25^	*	*	*	*		*	*		6/9
Lesiak et al.^26^	*	*		*	*	*	*		6/9
Dębińska et al.^28^	*	*	*	*		*	*		6/9
Zhang et al.^32^	*	*	*	*		*	*		6/9
Churnosov et al.^33^	*	*	*	*		*	*		6/9
Ercan et al.^34^	*	*	*	*		*	*		6/9
Dežman et al.^37^	*	*	*	*		*	*		6/9
Stemmler et al.^23^	*	*		*		*	*		5/9
Jurakic Toncic et al.^27^	*	*		*		*	*		5/9
Yu et al.^31^	*	*		*		*	*		5/9
Enomoto et al.^29^		*		*		*	*		4/9
González-Tarancón et al.^35^		*		*		*	*		4/9
Yoon et al.^36^		*		*		*	*		4/9
Kato et al.^22^				*		*	*		3/9
Woźniak et al.^38^				*		*	*		3/9

NOS: Newcastle-Ottawa Scale.

Studies were considered high quality if they scored 7 to 9 points, moderate quality if they scored 4 to 6 points, and low quality if they scored 0 to 3 points. A low overall score indicates a higher risk of bias in the study.

A descriptive data analysis was conducted. Two meta-analyses were performed: the first evaluated all genetic variants identified in the studies, and the second examined genetic variants at the level of individual studies. A dominance model was used to assess the influence of genetic variants as factors associated with odds of AD development.

The average age of patients across the studies was 15 years, whereas the average age of the controls was 23 years. Among patients evaluated for the presence of comorbidities associated with AD, 445 (59.4%) had asthma, 261 (34.9%) had rhinitis, and 43 (5.7%) had conjunctivitis. AD was mild in 1562 patients (44.6%), moderate in 1273 (36.4%), and severe in 535 (15.3 %).

**Figure 2.**
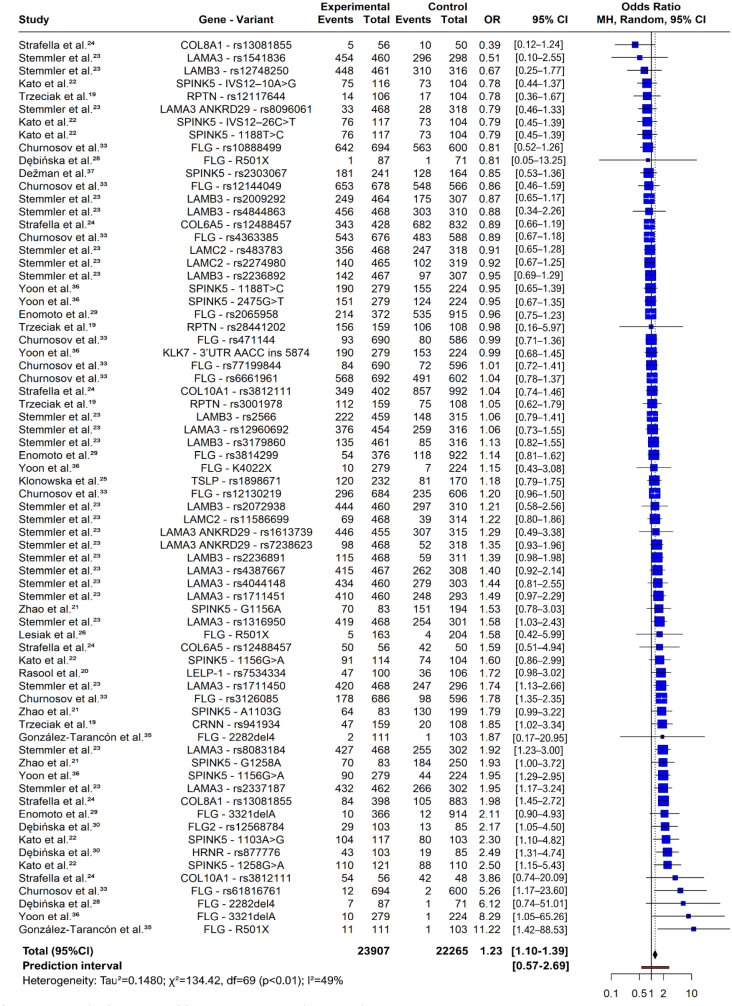
Forest-plot (meta-analysis by variant).

CI: confidence interval; df: degrees of freedom; MH: Mantel-Haenszel.

In the meta-analysis by variant, the random-effects model estimated the OR as 1.23 (95%CI 1.10–1.39, z=3.58, p=0.0003), indicating a significant association between genetic variants and AD development, as shown in the forest plot in Figure 2.^19,20,21,22,23,24,25,26,27,28,29,30,31,32,33,34,35,36,37,38^ The genetic variants mentioned in this review are reported in the forest plot using the nomenclature from their respective studies.

Heterogeneity among studies was moderate, with tau^2^ of 0.15 (95%CI 0.02–1.14), tau of 0.39 (95%CI 0.16–0.38), and I^2^ of 48.7% (95%CI 32.2–61.1%). The significant heterogeneity test (Q=134.42, degree of freedom (df)=69, p<0.0001) confirmed study variation.

There were no significant differences between the European and Asian subgroups (Q=0.36, df=1, p=0.5503). In the European group, the OR was 1.21 (95%CI 1.06–1.38), with tau^2^ of 0.15, tau of 0.38, and I^2^ of 48.6% (Q=97.26). In the Asian group, the OR was 1.31 (95%CI 1.05–1.64), with tau^2^ of 0.16, tau of 0.39, and I^2^ of 51.5% (Q=37.14).

The meta-analysis results suggest that the analyzed genetic variants may be associated with increased odds of AD development. The presence of heterogeneity should be considered, as environmental and multifactorial factors may have influenced these results.

**Figure 3.**
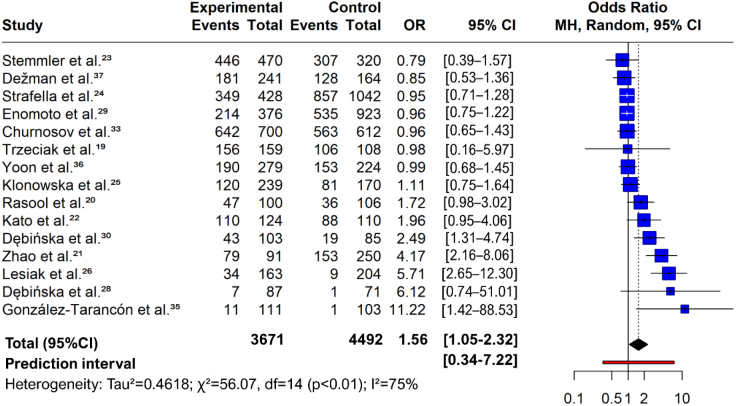
Forest-plot (meta-analysis by study).

CI: confidence interval; df; degrees of freedom; MH: Mantel-Haenszel.

In the meta-analysis by study, the random-effects model estimated an OR of 1.56 (95%CI 1.05–2.32, z=2.20, p=0.0277), indicating a significant association between genetic variants and AD development, as evidenced in the forest plot in Figure 3.^19,20,21,22,23,24,25,26,28,29,30,33,35,36,37^

The heterogeneity of studies was high, with tau^2^ of 0.46 (95%CI 0.13–1.37), tau of 0.68 (95%CI 0.35–1.17), and I^2^ of 75.0% (95%CI 58.7–84.9%). The heterogeneity test result was significant (Q=56.07, df=14, p<0.0001), confirming variation among the studies in the meta-analysis.

There was no significant difference between the European and Asian subgroups (Q=0.00, df=1, p=0.9758), indicating that subgroup variation did not significantly contribute to overall heterogeneity. In the European group, the OR was 1.57 (95%CI 0.89–2.77), with I^2^ of 73.9% (Q=34.51), whereas in the Asian group, it was 1.59 (95%CI 0.94–2.69), with I^2^ of 81.3% (Q=21.44).

Differences in population characteristics may explain the observed heterogeneity among the studies. Each study assessed a geographically and ethnically distinct population, which may have affected the results. Factors such as the prevalence of genetic variants, lifestyle, environmental factors, and access to healthcare varied significantly between regions and ethnic groups.

Based on the two meta-analyses, genetic variants were associated with increased odds of AD development. In the first meta-analysis, the variants R501X and 3321delA of *FLG* and A1103G, G1156A, and G1258A of *SPINK5* showed statistically significant results for increased odds of AD development. In the second meta-analysis, studies by Zhao et al.^21^, Lesiak et al.^26^, Dębińska et al.^30^, and González-Tarancón et al.^35^, reported statistically significant results regarding the odds of AD development, primarily by evaluating *FLG* and *SPINK5*. Both meta-analyses strongly suggested that variants of *SPINK5* and *FLG* may play a crucial role in AD development.

The heterogeneity among these studies should also be considered. Additionally, it is essential to note that the association between genetic variants and AD is complex and may have been influenced by factors such as gene-environment interactions.

## DISCUSSION

Atopic dermatitis is a multifactorial disease with phenotypic and genotypic heterogeneities. Variations in subtype, age, and severity interfere with research reproducibility. Genetic and molecular aspects, as well as variable prevalence among different ethnic groups, also contribute to the observed variations in AD.^39,40^ Recent large-scale studies integrating GWAS, QTLs, and functional assays have uncovered novel loci associated with skin barrier dysfunction and reaffirmed the pivotal role of genes such as *FLG* in the pathogenesis of AD.^41^

In this study, the average age of the patients was 15 years, whereas that of the controls was approximately 23 years. This difference could be explained by the higher prevalence of AD in children.^1^

Asthma was observed in 59.4% of patients with AD; 34.9% had allergic rhinitis, and 5.7% had conjunctivitis as a comorbidity. This association corroborate the concept of the “atopic march,” a progression of allergic diseases influenced by genetic, immunological, environmental, and epigenetic factors, as well as by skin barrier impairment.^8^ Starting in early childhood with AD, followed by the development of food allergies, asthma, and allergic rhinitis, this process is influenced by genetic, immunological, environmental, and epigenetic factors; skin barrier disruption; and alterations in the microbiome.^42,43,44^

Family history also plays an important role in this process. Children with atopic parents are at a higher risk of developing AD early in life. In addition, family history increases the risk of developing asthma.^45^ Children with AD are twice as likely to develop asthma than those without AD. The AD severity also influences this association; 70% of children with severe AD develop asthma. Children with late-onset AD (after 2 years of age) have a higher risk of developing allergic rhinitis, whereas those with early-onset AD (before 2 years of age) have a higher risk of developing asthma and allergen sensitization.^46,47^

Atopic dermatitis is heterogeneous, difficult to diagnose, and varies in its clinical presentation and severity. Symptom severity is assessed using scoring tools such as SCORing AD and Eczema Area and Severity Index, which aid in the treatment and monitoring of responses.^48^ The severity distribution varies, with a predominance of mild disease.^4^ The literature data corroborates the findings of this study, which identified a lower frequency of severe AD (15.3%) than that of milder forms of AD.

Both meta-analyses indicated that genetic variants were associated with increased odds of impaired skin barrier function in AD, highlighting the genetic importance of disease susceptibility. These findings are consistent with recent multiancestry analyses highlighting the relevance of barrier-related genetic variants and the importance of accounting for population diversity.^14^ While *FLG* and *SPINK5* are well established in AD etiology, our synthesis quantitatively confirms their impact across diverse populations and reveals significant associations of variants such as R501X and 3321delA in Spanish and South Korean cohorts, respectively. There was heterogeneity among the studies in both meta-analyses, suggesting variability in the effects of gene variants in different studies, possibly explained by demographic characteristics (geographic and ethnic diversities) and genetic and environmental factors.

In the meta-analysis by variants, the variants 2282del4, 3321delA, and R501X of *FLG* and A1103G, G1156A, T1188C, and G1258A of *SPINK5* were significant in some populations. This suggests a higher prevalence of these variants in certain regions, which influences disease susceptibility.

Subgroup analyses from both meta-analyses indicated no significant differences between the European and Asian populations. This suggests that the genetic variants related to AD do not follow a uniform pattern in these groups. Regional or local factors, such as environment, cultural habits, and gene–environment interactions, are likely to have contributed to these variations. The high heterogeneity in the subgroup analyses indicated substantial variations within each subgroup, possibly due to methodological differences, sampling variations, and genetic and environmental diversities within the continents.

Biomarker research, particularly in pediatric AD, holds promise for patient stratification, inflammation monitoring, and personalized treatment. Nonetheless, the inherent heterogeneity of AD, shaped by genetics, environment, microbiota, and methodological variability, demands integrative approaches that incorporate genomic, environmental, and microbial data.^5,15^

Genetic variations, mainly single-nucleotide polymorphisms, explain the physical differences among individuals. Approximately 85–90% of this variation occurs within a population, whereas only 10–15% occurs across all populations worldwide.^49^ These variations influence the applicability of genetic findings across different ethnic groups due to distinct genetic and environmental variations and gene-environment interactions.^50^

Research on human genetics has focused on populations of European descent, potentially perpetuating healthcare disparities by excluding certain population groups from clinically relevant discoveries.^49,51^ In this review, 60% of the studies originated in Europe, acknowledging this fact. Although there has been a recent expansion of studies involving Asian populations, individuals of other ancestries remain underrepresented and comprise less than 4% of the samples. Population stratification limits our understanding of disease biology because genetic associations identified in the European population may not apply to other groups, thereby hindering the discovery of new associations in other populations.^51,52^

Subgroup analyses did not reveal a consistent pattern of association between European and Asian populations. The observed heterogeneity likely reflects methodological variations and gene–environment interactions, reinforcing the need for further research involving underrepresented populations to validate and expand these genetic associations.^10,41^

Advances in DNA sequencing have enabled the exploration of human genetic variations, promoted a better understanding of AD genetic mechanisms and improving treatments.^53,54^ The findings of this review will pave the way for the development of new diagnostic and therapeutic tools for AD. Genetic studies can help identify individuals with higher odds of developing the disease, which is crucial for the development of prevention and early treatment strategies.

Beyond traditional genetic markers such as *FLG* and *SPINK5*, integrating multiomic data and developing polygenic risk scores may enhance the prediction of severe AD phenotypes and inform personalized treatment strategies, advancing the field of precision medicine in AD.^55^

Skin barrier mechanisms have been studied in AD, as they influence the disease course and severity. Skin barrier dysfunction has been linked to loss-of-function mutations in *FLG*, which lead to allergic sensitization and AD development in childhood, and may be associated with the later development of asthma and allergic diseases.^39,56^ Genetic alterations, especially in *FLG*, play a significant role in AD predisposition, with loss-of-function mutations found in approximately 10% of patients, which significantly influences its pathogenesis.^40,53^

The skin microbiome plays a key role in defending against pathogens and regulating local immune responses. In AD, reduced microbial diversity and increased colonization by Staphylococcus aureus have been associated with heightened inflammation and compromised skin barrier function.^10,57^

Located on chromosome 1q21, the *FLG* gene plays a central role in epidermal differentiation and carries loss-of-function variants associated with AD. These variants exhibit population-specific frequencies, such as R501X in European populations and 3321delA in Asian populations.^40,56^ In this review, significant associations were observed between the R501X variant in the Spanish population and the 3321delA variant in the South Korean population.

However, the relationship between AD and *FLG* is not direct, as reduced levels of filaggrin protein in the skin are observed independently of gene mutations,^58^ suggesting the influence of other factors that modulate the disease phenotype.

*SPINK5* on chromosome 5q32 encodes the serine protease inhibitor, lymphoepithelial Kazal-type inhibitor (LEKTI).^59^ Changes in skin pH lead to LEKTI dissociation, increasing kallikrein activity and resulting in skin desquamation and impaired skin barrier function.^60,61^

Loss-of-function mutations in *SPINK5* are associated with AD in some populations such as the Japanese population. Apart from AD, *SPINK5* variants are linked to Netherton Syndrome and atopic diseases such as asthma.^59,62^ This review observed that the prevalence of *SPINK5* variants and their associations with AD varied among ethnic groups, although they were more significant in Asian populations such as those in Japan and South Korea.

Other susceptibility loci associated with AD have been identified, particularly genes on chromosome 1q21, which are related to the epidermal differentiation complex.^63^ AD is influenced by various genetic factors that play crucial roles in the skin barrier function, and these genetic associations across different ethnic groups are essential for understanding the pathogenesis of AD.

The phenotypic and genotypic heterogeneity of AD, along with the ethnic variability in prevalence, could make the reproducibility of results and the generalization of conclusions. Additionally, the diversity of methods utilized in data collection in the articles and the underrepresentation of some populations in genetic studies limit the understanding of the relationship between genetic variants and AD. The presence of allergic comorbidities and the severity of the disease also influence the phenotypic expression of AD, making data analysis more complex.

By consolidating global data on barrier-related genetic variants, this review supports initiatives for risk stratification and future research on targeted interventions designed to restore skin barrier integrity and personalize AD management.^13^ Future studies with larger and more homogeneous samples should consider the interaction between genetic variants and environmental factors. Research that confirms and validates the results found in specific populations is necessary. Additionally, studies should focus on understanding how these factors contribute to the heterogeneity among populations and the development of personalized treatments.

Recent studies propose that integrating multiomic data with exposome analysis may overcome the limitations of isolated genetic approaches, enabling a more comprehensive understanding of AD and facilitating the development of biomarkers and targeted therapies.^13,41^

Several genetic variants were found to be significantly associated with AD, as evidenced by the present meta-analysis. Only those variants with an OR greater than 1 and CIs that did not cross the null value were considered significant, reinforcing the robustness of the findings.

Among the most representative, the *SPINK5* gene stands out due to the variantsA1103G, G1258A, and G1156A. Studies conducted by Kato et al.^22^ and Yoon et al.^36^ in Japanese and South Korean populations reported OR values ranging from 1.94 to 2.5. These variants affect the activity of the serine protease inhibitor LEKTI, which is essential for skin barrier homeostasis, supporting the structural role of the gene in AD pathogenesis.

The *LAMA3* gene, responsible for encoding one of the subunits of laminin-5 (laminin-332), also showed relevant associations through the variants rs1711450, rs8083184, and rs2337187, all identified in a study conducted by Stemmler et al.^23^ in Germany. The integrity of the dermoepidermal junction, which is heavily dependent on laminin-332, is a fundamental component in preventing the penetration of allergens and microorganisms, justifying its link to AD.

Other structural genes of the extracellular matrix also showed impact, such as *COL8A1*, through variant rs13081855, reported in a study by Strafella et al.^24^ Similarly, Dębińska et al.^30^ identified the *HRNR* variant rs877776 in Polish patients, with an OR of 2.48. Both reinforce the role of structural proteins in maintaining skin barrier integrity.

The *FLG* gene concentrated on the variants with the highest OR magnitudes. The rs3126085 (OR=1.78) and rs61816761 (OR=5.26) variants, observed in a Russian study by Churnosov et al.,^33^ along with the classical mutations R501X (OR=11.22) and 3321delA (OR=8.29), described in European populations by González-Tarancón et al.^35^ and Yoon et al.,^36^ support the substantial contribution of filaggrin as a predisposing factor. These variants impair corneocyte formation and water retention in the epidermis, facilitating the penetration of environmental allergens and microorganisms.

The identification of these variants, especially in epidermal barrier genes such as *FLG, SPINK5*, and *LAMA3*, has significant clinical implications. These variants may serve as genetic risk biomarkers for early-onset AD and underpin personalized medicine strategies, including neonatal screening, genetic counseling, and preventive approaches in individuals with a family history of the disease.

Nevertheless, some limitations should be acknowledged. This meta-analysis presented moderate to high heterogeneity (I^2^ ranging from 48.7 to 75%), indicating substantial variation among the included studies. These differences may be explained by geographic, ethnic, and methodological diversity, as most studies were conducted in European and Asian populations, with underrepresentation of African and Latin American groups, which restricts the generalizability of the findings. Moreover, although gene-environment interactions are known to play a crucial role in AD pathogenesis, many studies have not adequately controlled for environmental factors, which may have influenced the observed associations. Another important limitation is that different gene mutations were analyzed as distinct exposures within the same meta-analysis, despite all being related to the same outcome (skin barrier impairment in AD). This approach may have amplified heterogeneity, since variants differ in frequency, functional impact, and pathogenic potential. Finally, variability in sample sizes and in the inclusion and exclusion criteria across studies may also have affected comparability and interpretation of results.

This systematic review and meta-analysis confirms the strong association between FLG and SPINK5 variants and AD, while also highlighting additional genes such as LAMA3, HRNR, and COL8A1 that may contribute to skin barrier impairment. By integrating data from diverse European and Asian populations, the study provides consistent evidence that genetic variants affecting barrier function significantly increase susceptibility to AD. These findings support translational strategies such as early risk stratification, genetic counseling, and personalized treatment approaches.

In conclusion, this review indicates that genetic variants are significantly associated with susceptibility to AD and highlights the importance of genetic diversity and gene–environment interactions in epidermal barrier dysfunction observed in AD.

## Data Availability

The database that originated the article is available with the corresponding author.
